# Computer-aided optimal design for flexible cable in aerospace products based on dynamic analogy modeling

**DOI:** 10.1038/s41598-022-09880-9

**Published:** 2022-04-06

**Authors:** Hongwang Du, Qinwen Jiang, Wei Xiong

**Affiliations:** Ship Electromechanical Equipment Institute, Room 315, Mechanical and Electrical Building, Dalian Maritime University, No. 1 Linghai Road, Ganjingzi District, Dalian, 116026 Liaoning China

**Keywords:** Aerospace engineering, Engineering, Mechanical engineering

## Abstract

Due to large, complex deformations, the accurate design of cables has become a major problem in the manufacturing of aerospace products. The current design method often leads to large products, uncertain centroids, and poor reliability. To solve these problems, a computer-aided optimal design method for flexible cables was proposed based on dynamic analogy modeling. A nonlinear optimization model was established by combining Cosserat theory and the minimum potential energy principle. The total deformation energy was considered as the optimization object, and Euler parameters were used as control variables to describe the cable geometric shape. Considering the length and bending radius requirements, the normalized form of the cable constraints was expressed by the cross-section position and orientation matrix. An efficient method to solve this problem using finite element discretization and the primitive dual interior point method was proposed. A digital wiring module was developed based on an open source geometry kernel system, and a cable geometry test bench was built. To verify our model, a satellite wiring simulation example was implemented using the module, SolidWorks, and the test bench. Our method achieved the optimal design for the cable length and geometric shape. A theoretical and technical foundation for effectively solving the problem of large cable manufacturing errors and realizing the lightweight design of aerospace products was outlined.

## Introduction

In aerospace products like satellites, cables are laid out like human neurons. Cables play the role of transmitting information and energy and possess a large proportion of aerospace products. Cable design is an important factor that affects the quality of the whole aerospace product manufacturing process^1^. In the earliest method, physical prototypes or models were formed, after which the cable was installed repetitively until the requirements were met. This method has a long cycle time and high cost and has been phased out gradually^2^.

With the development of CAD (computer-aided design) technology, computer-aided wiring technology has developed rapidly. Current commercial CAD modeling software packages all supply a wiring design module. The basic idea of wiring in any software is to express the cable shape in the form of a spline curve according to key connection points, which are determined by electrical schematic diagrams^3^. This method may use two-dimensional (2D) or three-dimensional (3D) designs. The design idea is closer to reality and can account for certain spatial constraints. However, it often leads to large cable length errors because the complex large deformation characteristics are ignored. Worse yet, other problems may appear, such as large product mass, uncertain centroids, and poor reliability arise due to the inconsistencies between the actual wiring shape and the design model. These problems are inconsistent with the concept of intelligent manufacturing proposed by Industry 4.0.

To solve these problems, computer-aided optimal design for flexible cables should be based on accurate mechanical modeling. Wiring methods with physical modeling can provide accurate length design and routing paths, realize lightweight product design, and ensure product quality. Computer-aided optimal design is a type of digital design. Wiring is performed based on physical modeling in a virtual environment. The basis of the wiring is a digital prototype and electrical schematic. Finally, a reasonable and reliable cable design scheme can be obtained with determined cable design parameters, including the length, path, branch location, and binding fixation scheme. During the whole process, the cable design is completely consistent with the actual situation, thereby avoiding the production of physical prototypes and changing the design concept^4^. Thus, research of cable digital design involves the integration of many complex aspects, including describing the large deformation, physical modeling, numerical calculations, and complex geometric shape expression. The key technologies are physical modeling and numerical calculations.

Modeling of cable digital designs is a static problem. We must solve the cable geometry under multiple constraints to obtain the design parameters. Because of the large deformation, physical modeling and calculations for cables are very challenging. As a kind of one-dimensional flexible body (1DFB), cables have similar mechanical properties to those of ropes, hairs, hoses, DNA molecular chains, and branches. Since the 1990s, a variety of mechanical modeling methods have emerged.

In the early days, spline curves were often used to describe the geometric shape of a 1DFB because of the geometric similarity between a 1DFB and a spline curve. To express it vividly, the spline curves were endowed with mechanical properties. Celniker et al. proposed the concept of an energy curve, in which the deformation energy is expressed by the combination of mechanical factors and spline curves. The equilibrium state satisfies the principle of minimum potential energy to determine the geometric shape of a 1DFB^5^. Liu et al. used a spline curve to do the assembly process planning. The cable was considered to be a central line with a certain radius; the line was fit by non-uniform B-spline curves, and the cable balanced state was solved by energy optimization^6^. Menon et al. proposed a new paradigm for the simulation and rendering of the motion of 1DFBs. The motion of 1DFBs, represented using splines, is computed using a tractrix-based approach. The tractrix based approach yielded a more natural and realistic motion, with the motion decreasing along the length of 1DFBs^7^. However, the spline curve could only reflect the flexibility of the cable partly and could not handle torsion. Thus, its accuracy was poor, and it could not be used for cable computer-aided optimal design.

The mass–spring method was the second simplified method used to express the geometric shape of 1DFBs. The principle was that a cable could be discretized into a series of mass points and springs between adjacent points, after which the geometric shape could be obtained through the Newton force principle. This method was first used in cloth simulations and then was introduced into cable modeling^8^. The early mass–spring method only considered the tension and bending deformation. After a period of development, scholars added a torsion spring between adjacent springs to reflect the torsion^9^. Selle et al. used this method for hair simulation^9^. Lv et al. used a similar model to simulate cable assembly process planning^10^. The mass–spring method could partly describe the deformation of a 1DFB and had a high calculation efficiency. However, this method possesses two problems for cable computer-aided optimal design: it is difficult to describe complex constraints, such as clamps, holes, and branches, and the linear spring torsion description is quite inaccurate, because cable torsion is nonlinear.

The finite element method (FEM) can describe the cable using geometric meshes. Interpolation functions are used to express the deformation and stress of each mesh, and the cable macroscopic deformation can be obtained^11^. This method can avoid the appearance of divergent series and produce more accurate calculations of physical properties, such as the cable weight and stiffness. However, it requires a large number of discrete meshes, which is difficult to achieve with the real-time requirement of 1DFB simulations. Therefore, this method is usually combined with other mechanic models to ensure efficiency and accuracy simultaneously.

Nonlinear mechanics of elastic rods are a special model for the analysis of the deformation characteristics of 1DFBs, and the classic model is named Cosserat theory. Its principle is that the spatial geometric shape can be expressed by the translation and rotation of the cross-section relative to the centerline, assuming the cross-section is rigid. The equilibrium problem of elastic rods becomes a discrete system with the arc coordinate s as the independent variable^12^. Pai et al. applied Cosserat theory to model a suture surgical line and proposed a quasi-static method of two-phase integration to solve the problem. However, this model was only applicable to cases with small deformation, and the accuracy of the numerical integration could not be guaranteed for large deformation^13^. Grégoire et al. used this model to route cables in a virtual environment, considering the weight and friction. The cable equilibrium equations were derived by the variational energy principle and Newton's second law, containing bending and torsion but no stretching. This approach could achieve basic wiring and shape simulation but it neglected the cable length design and contact constraint. Moreover, to ensure real-time performance, the mechanical prototype was equivalent to a spring, which resulted in a low solution accuracy^14^. Linn et al. modeled flexible cables based on Cosserat theory and simulated the quasi-static deformation of cables in sufficiently slow motion^15^. Furthermore, they presented a mechanical simulation method of 1DFBs and applied it to cable assembly process planning for automotive engines. They established Euler–Lagrange differential equations based on Cosserat theory and solved them with a standard differential equation solver. Although this method exhibited a high efficiency, large deformation and complex collision constraints required further verification^16^. Spillmann et al. proposed a “CORDE” model for dynamic interactive simulations of the 1DFB based on Cosserat theory. The dynamic deformation process could be obtained by numerical integration of the Lagrangian motion equation^17^. Hermansson et al. carried out cable geometric shape simulations of car doors based on Cosserat theory. The model considered the position constraints of the cable connectors and used a variational energy principle. However, this method was unable to handle complex constraints, such as clamps, on the cables^18^. Lv et al. used Cosserat theory to simulate the assembly process of multi-branch cables. The potential energies of joints were calculated by taking the topology and anatomical features into consideration. The configuration of the cable was then calculated by minimizing its potential energy^19^.

In summary, the nonlinear mechanics of elastic rods can describe the complex spatial deformation shape of 1DFBs. This mechanical idea can account for bending and torsion and can even simultaneously model the tension and shear deformation of the 1DFBs comprehensively and accurately. Compared with other methods, it is most suitable for cable modeling. For this method, statics modeling has two methods: One is based on classical mechanics, in which the sum of all forces and moments in the constrained flexible cable system is zero, and the other is based on variational and functional analysis, where the constrained flexible cable system satisfies the principle of minimum potential energy in a stable equilibrium state. The Cosserat basic theory has matured. However, there are still several problems to be solved urgently if this theory is to be used in computer-aided optimal design. The first issue is how to express the complex constraints of a cable by appropriate mathematical formulas, such as clamps, collision contacts, surfaces, and through-holes. The second issue is how to solve the problem accurately and efficiently to obtain the cable design length and final wiring geometric shape.

This study focuses on the computer-aided optimal design of flexible cables, similar to the research in Ref.^19,20^. Both described the energy of cable deformation from the point of view of analytical mechanics based on Cosserat theory. In the former, the cable force based on the energy was deduced and the equilibrium equation based on Newton's law of mechanics was established. In the latter, the equilibrium shape of branch cables was simulated using the idea of nonlinear optimization based on the principle of minimum potential energy. In both studies, only the geometric shape was simulated, but the design parameters, such as the cable length and bending radius, were not optimized from the point of view of product optimization design. To solve this problem, a novel method based on the Cosserat theory is proposed herein to realize the computer-aided optimal design of flexible cables. An energy model to describe the cable balance is established in Sect. 2 based on a variational principle, which accounts for the complex constraints and requirements of the length and bending radius. An efficient large-scale nonlinear optimization algorithm is introduced in Sect. 3 to obtain a reasonable length, bending radius, and spatial geometry of the cable. A flexible cable wiring module is developed in Sect. 4. A satellite wiring simulation example is provided in Sect. 5, which was implemented using the module, SolidWorks, and the test bench to verify our method. Finally, the study is concluded.

## Static modeling

### Cable spatial geometric shape description

**Figure 1:**
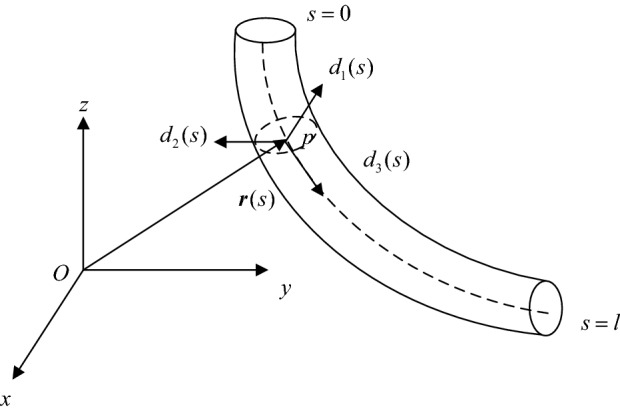
Spatial geometric shape of cable.

Based on dynamic analogy method, the spatial geometric shape of the cable is formed by displacement and rotation of a cross-section along the centerline^21,22^. Therefore, the geometric shape can be expressed by the cross-section orientation and the shape of the centerline, as shown in Fig. 1.

The cable geometric shape is expressed by the variables (**r**(s), (**d**_1_(s), **d**_2_(s), **d**_3_(s)) and can be identified by **r**(s) and **C**(**d**_1_(s), **d**_2_(s), **d**_3_(s)). **r**(s) represents the shape of the centerline. **C**(**d**_1_(s), **d**_2_(s), **d**_3_(s)) is a directional cosine matrix formed by the projection of the moving coordinate system *P*-*d*_1_*d*_2_*d*_3_ relative to the fixed coordinate system *O*-*xyz* and represents the cross-section orientation.

Euler parameters are introduced to describe the directional cosine matrix. Their physical meaning is that the arbitrary rotation of a rigid body around a fixed point *O* can be realized by a single rotation (angle *χ*) of a rotation axis P(p_1_, *p*_2_, *p*_3_) around that point. This rotation cosine matrix **C** could be expressed by Eq. (1)^23^. Compared with Euler angles, the Euler parameters can effectively avoid singularities and are suitable for expressing large deformations of cables:

1 $$ {\mathbf{C}} = \left( {\begin{array}{*{20}c} {p_{1}^{2} (1 - \cos \chi ) + \cos \chi } & {p_{2} p_{1} (1 - \cos \chi ) - p_{3} \sin \chi } & {p_{3} p_{1} (1 - \cos \chi ) + p_{2} \sin \chi } \\ {p_{2} p_{1} (1 - \cos \chi ) + p_{3} \sin \chi } & {p_{2}^{2} (1 - \cos \chi ) + \cos \chi } & {p_{3} p_{2} (1 - \cos \chi ) - p_{1} \sin \chi } \\ {p_{3} p_{1} (1 - \cos \chi ) - p_{2} \sin \chi } & {p_{3} p_{2} (1 - \cos \chi ) + p_{1} \sin \chi } & {p_{3}^{2} (1 - \cos \chi ) + \cos \chi } \\ \end{array} } \right). $$

The Euler parameters are quaternions, and the definition of quaternions are expressed as follows:

2 $$ q_{1} = \cos \frac{\chi }{{2}},q_{2} = p_{1} \sin \frac{\chi }{{2}},q_{3} = p_{2} \sin \frac{\chi }{{2}},q_{4} = p_{3} \sin \frac{\chi }{{2}}\; $$

The four symbols *q*_1_, *q*_2_, *q*_3_, *q*_4_ are the Euler parameters, and the directional cosine matrix of *P*-*d*_1_*d*_2_*d*_3_ relative to *O*-*xyz* can be represented by the Euler parameters as follows:

3 $$ C{ = }\left( {\begin{array}{*{20}c} {2\left( {q_{1}^{2} + q_{2}^{2} } \right) - 1} & {2\left( {q_{2} q_{3} - q_{1} q_{4} } \right)} & {2\left( {q_{2} q_{4} + q_{1} q_{3} } \right)} \\ {2\left( {q_{2} q_{3} + q_{1} q_{4} } \right)} & {2\left( {q_{1}^{2} + q_{3}^{2} } \right) - 1} & {2\left( {q_{3} q_{4} - q_{1} q_{2} } \right)} \\ {2\left( {q_{2} q_{4} - q_{1} q_{3} } \right)} & {2\left( {q_{3} q_{4} + q_{1} q_{2} } \right)} & {2\left( {q_{1}^{2} + q_{4}^{2} } \right) - 1} \\ \end{array} } \right). $$

A twisting vector named **ω** is introduced to express the cable bending and torsion deformation, and its three component expressions in *P*-*d*_1_*d*_2_*d*_3_ are shown as follows:

4 $$ {{\varvec{\upomega}}}{ = }\omega_{1} {\mathbf{d}}_{1} + \omega_{2} {\mathbf{d}}_{2} + \omega_{3} {\mathbf{d}}_{3} . $$

The relationships between each coordinate of *P*-*d*_1_*d*_2_*d*_3_ and **ω** are as follows:

5 $$ \begin{array}{*{20}l} {\frac{{d{\mathbf{d}}_{k} }}{ds} = {{\varvec{\upomega}}} \times {\mathbf{d}}_{k} \;\;\;k = 1,2,3} \hfill \\ {{\text{d}}_{3} = {\text{d}}_{1} \times {\text{d}}_{2} } \hfill \\ {{\text{d}}_{k} \times {\text{d}}_{k} = 0\;\;\;k = 1,2,3} \hfill \\ {{\text{d}}_{k} \; \cdot \;\;{\text{d}}_{k} \; = 1\;\;\;k = 1,2,3} \hfill \\ {{\text{d}}_{1} \times {\text{d}}_{2} = {\text{d}}_{2} \times {\text{d}}_{3} = {\text{d}}_{3} \times {\text{d}}_{1} = 0} \hfill \\ \end{array} . $$

According to Eqs. (3)–(5), the following relationships between **ω** and the Euler parameters can be deduced:

6 $$ \begin{array}{*{20}c} {\omega_{1} = 2\left( { - q_{2} q^{\prime}_{1} + q_{1} q^{\prime}_{2} + q_{4} q^{\prime}_{3} - q_{3} q^{\prime}_{4} } \right)} \\ {\omega_{2} = 2\left( { - q_{3} q^{\prime}_{1} - q_{4} q^{\prime}_{2} + q_{1} q^{\prime}_{3} + q_{2} q^{\prime}_{4} } \right)} \\ {\omega_{3} = 2\left( { - q_{4} q^{\prime}_{{1}} + q_{{3}} q^{\prime}_{{2}} - q_{2} q^{\prime}_{3} + q_{1} q^{\prime}_{4} } \right)} \\ \end{array} . $$

### Finite element discretization

**Figure 2:**
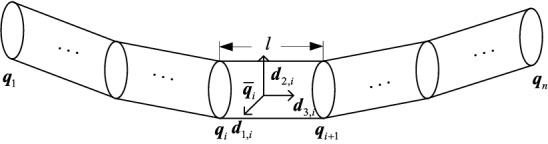
Finite element discretization for cable.

The cable is discretized into nodes and elements based on the finite element method. As shown in Fig. 2, the cable is divided into *n* nodes along the centerline and *n*-1 elements.

The cable stretching stiffness is very large, and thus, the stretching deformation is neglected. The distance between adjacent nodes are represented by a constant with the symbol *l*. The Euler parameters are located at the cross-section of the node, and the Euler parameters are unchanged in the element. By linear interpolation in the element, the Euler parameters and derivatives in any element i can be obtained, as shown in Eqs. (7) and (8):

7 $$ q^{\prime}_{i} \approx \frac{1}{l}\left( {q_{i + 1} - q_{i} } \right), $$

8 $$ \overline{{q_{i} }} = \frac{1}{2}\left( {q_{i} + q_{i + 1} } \right). $$

The position of element *i* is denoted by r($\overline{\mathrm{q}}$_*i*_), and the orientation of element *i* is denoted by C_*i*_(*d*_1,*i*_(s), *d*_2,*i*_(s), *d*_3,*i*_(s)). The expression of the element position and orientation by the discrete Euler parameters is realized by Eqs. (3) and (5), expressed as follows:

9 $$ r_{i} = \left( {\begin{array}{*{20}c} {r_{1,i} } \\ {r_{2,i} } \\ {r_{3,i} } \\ \end{array} } \right) = \left( {\begin{array}{*{20}c} {r_{1,1} + \sum\limits_{j = 0}^{i} {2l(\overline{q}_{2,j} \overline{q}_{4,j} + \overline{q}_{1,j} \overline{q}_{3,j} )} } \\ {r_{2,1} + \sum\limits_{j = 0}^{i} {2l(\overline{q}_{2,j} \overline{q}_{4,j} + \overline{q}_{1,j} \overline{q}_{3,j} )} } \\ {r_{3,1} + \sum\limits_{j = 0}^{i} {l(2(\overline{q}_{1,j}^{2} + \overline{q}_{4,j}^{2} ) - 1)} } \\ \end{array} } \right). $$

Combining Eqs. (3) and (9), the directional cosine matrix of element *i* can be expressed as follows:

10 $$ C_{i} { = }\left( {\begin{array}{*{20}c} {2\left( {\overline{q}_{1,i}^{2} + \overline{q}_{2,i}^{2} } \right) - 1} & {2\left( {\overline{q}_{2,i} \overline{q}_{3,i} - \overline{q}_{1,i} \overline{q}_{4,i} } \right)} & {2\left( {\overline{q}_{2,i} \overline{q}_{4,i} + \overline{q}_{1,i} \overline{q}_{3,i} } \right)} \\ {2\left( {\overline{q}_{2,i} \overline{q}_{3,i} + \overline{q}_{1,i} \overline{q}_{4,i} } \right)} & {2\left( {\overline{q}_{1,i}^{2} + \overline{q}_{3,i}^{2} } \right) - 1} & {2\left( {\overline{q}_{3,i} \overline{q}_{4,i} - \overline{q}_{1,i} \overline{q}_{2,i} } \right)} \\ {2\left( {\overline{q}_{2,i} \overline{q}_{4,i} - \overline{q}_{1,i} \overline{q}_{3,i} } \right)} & {2\left( {\overline{q}_{3,i} \overline{q}_{4,i} + \overline{q}_{1,i} \overline{q}_{2,i} } \right)} & {2\left( {\overline{q}_{1,i}^{2} + \overline{q}_{4,i}^{2} } \right) - 1} \\ \end{array} } \right). $$

Combined with Eqs. (6) and (10), the twisting vector of element *i* can be expressed as follows:

11 $$ \begin{array}{*{20}c} {\omega_{1,i} = 2\left( { - \overline{q}_{2,i} q^{\prime}_{1,i} + \overline{q}_{1,i} q^{\prime}_{2,i} + \overline{q}_{4,i} q^{\prime}_{3,i} - \overline{q}_{3,i} q^{\prime}_{4,i} } \right)} \\ {\omega_{2,i} = 2\left( { - \overline{q}_{3,i} q^{\prime}_{1,i} - \overline{q}_{4,i} q^{\prime}_{2,i} + \overline{q}_{1,i} q^{\prime}_{3,i} + \overline{q}_{2,i} q^{\prime}_{4,i} } \right)} \\ {\omega_{3,i} = 2\left( { - \overline{q}_{4,i} q^{\prime}_{{{1},i}} + \overline{q}_{{{3},i}} q^{\prime}_{{{2},i}} - \overline{q}_{2,i} q^{\prime}_{3,i} + \overline{q}_{1,i} q^{\prime}_{4,i} } \right)} \\ \end{array} . $$

### Minimum potential energy principle

Actually, the cable routing design method is to solve the spatial geometric shape in an equilibrium state. According to the minimum potential energy principle of elastic mechanics, the real displacement makes the total potential energy of an elastomer take a stationary value, that is, the first-order variation is zero^23^. Therefore, by solving the minimum value problem of the total potential energy of the cable under the boundary condition, the displacement of all the cross-sections with the minimum potential energy can be obtained, and the cable geometric shape in the equilibrium state can be obtained.

The total potential energy of the cable is composed of the elastic potential energy produced by elastic strain and gravity. The elastic potential energy is generated by the relative position and orientation change of each cable segment element and is equal to the sum of the bending and torsion potential energies. The gravity potential energy is evenly distributed in each cable element, which is related to the element location. Thus, the total potential energy of the cable can be described by discrete Euler parameters as follows:

12 $$ E = \sum\limits_{i = 1}^{n - 2} {E_{bending,i} } { + }\sum\limits_{i = 1}^{n - 2} {E_{torsion,i} } { + }\sum\limits_{i = 1}^{n - 1} {E_{weight,i} } . $$

### Cable bending potential energy

**Figure 3:**
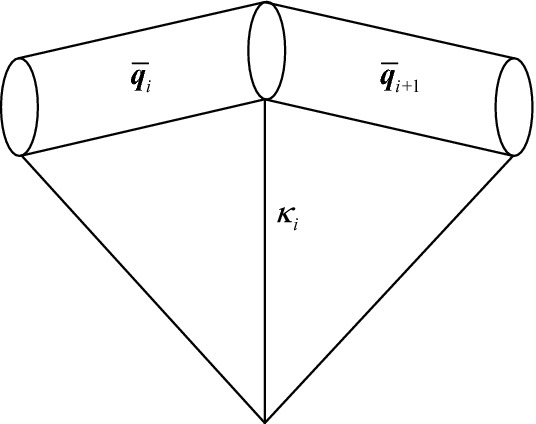
Bending deformation of adjacent elements.

As shown in Fig. 3, bending deformation is caused by the angle change between two adjacent elements.

The curvature radius is equal to the radius of the circumferential circle of the triangle formed by three continuous nodes. The cable bending potential energy of element *i* and *i* + 1 E_*bending,i*_ could be calculated as follows:

13 $$ E_{bending,i} = \frac{1}{2}K_{b} \kappa_{i}^{2} . $$

The curvature *κ*_*i*_ in Eq. (13) can be calculated as follows:

14 $$ \kappa_{i}^{2} = \omega_{1,i}^{2} { + }\omega_{2,i}^{2} . $$

The bending stiffness *K*_*b*_ can be calculated as follows:

15 $$ K_{b} { = }\iint\limits_{A} {Ex^{2} }dA = E\frac{{\pi R^{4} }}{{4}}, $$

where *E* is the elastic modulus of the cable material, and *R* is the radius of the cross-section.

### Cable torsion potential energy

**Figure 4:**
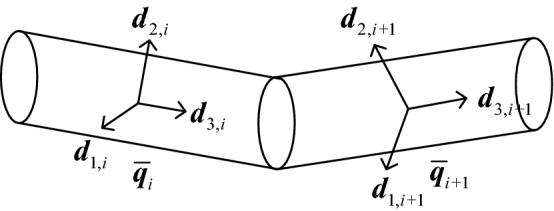
Torsion deformation between adjacent elements.

The cable torsion deformation consists of a Frenet torsion and a pure material torsion. The Frenet torsion is defined by the geometrical torsion of the centerline, and the pure material torsion shows the position of the material lines relative to the Frenet frames. According to Eq. (5), the cable torsion potential energy is a function of the third twisting vector component *ω*_3_. As shown in Fig. 4, cable torsion can be expressed by the Euler parameters of adjacent elements, and the torsion potential energy of element *i* can be expressed as follows:

16 $$ E_{torsion,i} = \frac{1}{2}K_{t} \omega_{3,i}^{2} . $$

The torsion stiffness *K*_*t*_ can be calculated as follows:

17 $$ K_{t} { = }\iint\limits_{A} {Gr^{2} }dA = G\frac{{\pi R^{4} }}{2}, $$

where *G* is the shear modulus of the cable material.

### Cable weight potential energy

**Figure 5:**
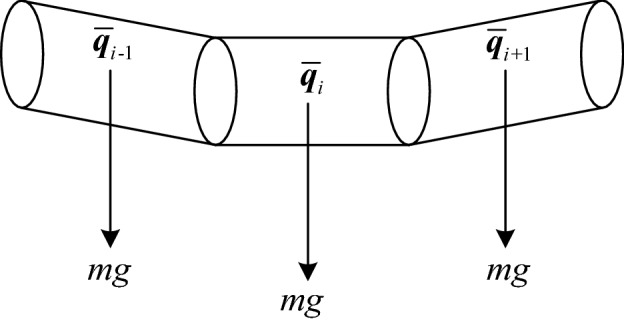
Element weight.

Gravity, as an external force with a constant direction acting on the cable, has a great influence on the cable deformation. The longer the cable is, the more significant its influence becomes. As shown in Fig. 5, the cable length of each element is denoted as *l*, gravitational acceleration is denoted as *g*, and the mass of each element is denoted as *m*. Thus, the total cable mass is (*n*-1)*m*.

The cable weight potential energy of element *i* can be expressed as follows:

18 $$ E_{weight,i} = mg\;l\left( {2\left( {\overline{q}_{{{1},i}}^{{2}} + \overline{q}_{{{4},i}}^{{2}} } \right) - {1}} \right). $$

The mass of each element can be calculated as follows:

19 $$ m{ = }\rho \pi R^{2} l, $$

where *ρ* is the cable density.

## Mathematic model solving

### Optimization problem description

In the above section, the spatial geometric shape and potential energy equations of the cable were established, taking the Euler parameters as reference variables. Based on the minimum potential energy principle, the cable spatial geometric shape can be determined by solving the Euler parameters in the equilibrium state. Therefore, the Euler parameters were chosen as the decision variables. For different spatial constraints, Euler parameters of different cross-sections have different forms. The optimization problem of the cable static model is described as follows:

Decision variables:

20 $$ q_{k,j} \in {\mathbb{R}}^{4n} ,\;(k = 1,2,3,4;j = 1,2, \cdots ,n), $$

Optimization objective:

21 $$ E = \sum\limits_{i = 1}^{n - 2} {E_{bending,i} ({\mathbf{q}}_{i} )} { + }\sum\limits_{i = 1}^{n - 2} {E_{torsion,i} ({\mathbf{q}}_{i} )} { + }\sum\limits_{i = 1}^{n - 1} {E_{weight,i} ({\mathbf{q}}_{i} )} , $$

Constraint conditions:

22 $$ f_{j} \left( {\mathbf{q}} \right) = q_{1,j}^{2} + q_{2,j}^{2} + q_{3,j}^{2} + q_{4,j}^{2} - 1 = 0,\;\;\;\;\;j = 1,2, \cdots ,n\,, $$

23 $$ \begin{array}{*{20}c} {g_{j} \left( {\mathbf{q}} \right) = {\varvec{r}}_{k} ({\mathbf{q}}_{j} ) - {\varvec{r}}_{j} = 0,\;\;\;\;\;\;\;k = 1,2,3} \\ {h_{j} \left( {\mathbf{q}} \right) = {\varvec{d}}_{k} ({\mathbf{q}}_{j} ) - {\varvec{d}}_{j} = 0,\;\;\;\;\;\;k = 1,2,3} \\ \end{array} , $$

24 $$ \kappa_{i}^{2} = \omega_{1,i}^{2} { + }\omega_{2,i}^{2} \le \kappa_{0} , $$

where *i* represents a node, *j* represents an element, *k* represents a component of a vector, and *κ*_0_ is the curvature radius requirement, which is determined by the minimum curvature radius.

For a cable with *n* nodes, there are *n* − 1 elements, *n* − 2 bending and torsion potential energy formulas, and *n* − 1 weight potential energy formulas. The above Euler parameters standardization constraint *f*_*j*_(**q**) is an internal constraint of the model, and the modulus of the Euler parameters is 1. To normalize the form of the different cable constraints, cross-sectional position and orientation matrices are proposed, denoted as *g*_*j*_(**q**) and *h*_*j*_(**q**). The significance of this expression is that, regardless of the constraints, such as boundaries, clamps, through-holes, and contacts, the mathematical expressions of the constraints have specific positions and orientations at different cross-sections. The position and orientation can be expressed using the Euler parameters. Compared to the methods presented in Ref.^19,20^, this method of constraint processing is simpler, eliminating the need to derive different mathematical formulas for different constraints.

### Algorithmic design

**Figure 6:**
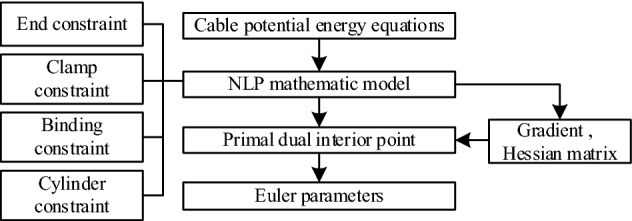
Flow chart of the optimization problem.

The optimization model described in the previous section contains both equality and inequality constraints, comprising a nonlinear multi-dimensional optimization problem. We propose a solution algorithm based on the primal–dual interior point method. Firstly, the Euler parameters of each discrete section are obtained. The position and attitude of each section along the arc length of the cable are subsequently calculated according to Eqs. (9) and (10). The flow chart of the algorithm is shown in Fig. 6.

### Solving optimization problem

The nonlinear multi-dimensional optimization problem with equality and inequality constraints is one of the most difficult optimization problems in mathematical programming. In this study, the constraints of the static optimization problem are very large, and the equality constraints are nonlinear. To solve this with appropriate accuracy and efficiency, we propose a primal dual-interior point method as the solution algorithm.

The standard form of the nonlinear optimization problem described in Eqs. (20)–(24) is expressed as follows:

25 $$ \left\{ \begin{gathered} \min f\left( x \right) \hfill \\ s.t.\;c\left( x \right) = 0\; \hfill \\ \;\;\;\;\;x \ge 0 \hfill \\ \end{gathered} \right.. $$

For Eq. (25), if a point x^*^ ∈ ℝ satisfies all the constraints, then x^*^ is called feasible point. All the constraints form boundaries. The Euclidean space in which all feasible points are located is called the feasible region. The points at which all feasible points lie at the boundary are called boundary points. For equality constraints, all feasible points are boundary points.

**Figure 7:**
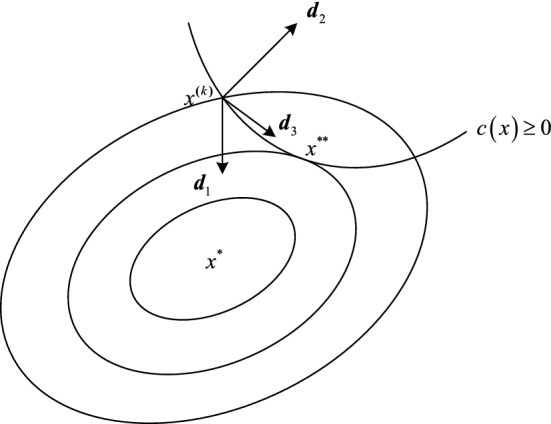
Feasible direction and descending direction.

The determination of the search direction is the key to solving an optimization problem. The feasible direction and descending direction are two important search directions. The former is the direction in which the iteration point satisfies the constraints in the search direction, while the latter is the direction in which the iteration point makes the objective function descend in the search direction. For our optimization problem, the restrictions on the search direction are shown in Fig. 7.

At the iteration point *x*^(*k*)^, the search direction **d**_1_ is originally the feasible descent direction of the constraint condition but becomes the infeasible direction under the constraint c(*x*) ≥ 0; both **d**_2_ and **d**_3_ are feasible directions of the constraint c(*x*), where **d**_3_ is the feasible descent direction.

Based on the designed feasible descent algorithm, the primitive dual-interior point method can be used to solve Eq. (25). The algorithm steps are described below. More detailed information can be found elsewhere^24^:

1. According to the constraints, a function is constructed and added to the objective function. The new function constructed is called a Lagrange function, expressed as follows:
  26 $$ \min \varphi_{\mu } \left( x \right){ = }f\left( x \right) - \;\mu \sum\limits_{i = 1}^{n} {\;\ln \left( {x^{(i)} } \right)} , $$
  where *μ* is the obstacle factor. The interior-point method solves the nonlinear optimized Karush–Kuhn–Tucker equation as follows:
  27 $$ \left\{ \begin{gathered} \nabla f(x) + \nabla c\left( x \right)\lambda - z = 0 \hfill \\ c\left( x \right) = 0 \hfill \\ {\text{X}} {\text{Z}}_{e} - \mu e = 0 \hfill \\ \end{gathered} \right., $$
  where ▽*f*(*x*) is the derivative of the objective function, ▽*c*(*x*) is the Jacobian matrix of the equality constraint, λ and *z* are the Lagrangian multiplier and the dual variable, respectively, **X** and **Z** represent the diagonal matrix, and *x* represents the diagonal elements.
2. Equation (27) is linearized using the damped Newton method. The linear equation that is set along the direction (*d*_*k*_^*x*^, *d*_*k*_^*λ*^, *d*_*k*_^*z*^) at the iteration point (*x*_*k*_, *λ*_*k*_, *z*_*k*_) is expressed as follows:
  28 $$ \left[ {\begin{array}{*{20}c} {W_{k} } & {A_{k} } & { - I} \\ {A_{k}^{T} } & 0 & 0 \\ {Z_{k} } & 0 & X \\ \end{array} } \right]\left( {\begin{array}{*{20}c} {d_{k}^{x} } \\ {d_{k}^{\lambda } } \\ {d_{k}^{Z} } \\ \end{array} } \right) = - \left( {\begin{array}{*{20}c} {\nabla f(x_{k} ) + A_{k} \lambda_{k} - z_{k} = 0} \\ {c(x_{k} )} \\ {X_{k} Z_{k} - \mu_{j} e} \\ \end{array} } \right), $$
  where *W*_*k*_ is the Hessian matrix of the original Lagrangian function *φ*, and *A*_*k*_ is the Jacobian matrix of the equality constraint. The elimination of the search direction of the inequality-constrained multipliers can be obtained using the following equations:
  29 $$ \left( {\begin{array}{*{20}c} {W_{k} + X_{k}^{ - 1} Z_{k} } & {A_{t} } \\ {A_{k}^{T} } & 0 \\ \end{array} } \right)\left( {\begin{array}{*{20}c} {d_{x}^{k} } \\ {d_{x}^{\lambda } } \\ \end{array} } \right) = - \left( {\begin{array}{*{20}c} {\nabla \varphi_{u} (x_{k} ) + A_{k} \lambda_{k} } \\ {c(x_{k} )} \\ \end{array} } \right). $$
3. After obtaining the search direction, the new iteration point is defined as follows:
  30 $$ \begin{array}{*{20}c} {x_{k + 1}^{{}} = x_{k}^{{}} + \alpha_{k} d_{k}^{x} } \\ {\lambda_{k + 1}^{{}} = \lambda_{k}^{{}} + \alpha_{k} d_{k}^{\lambda } } \\ {z_{k + 1}^{{}} = z_{k}^{{}} + \alpha_{{_{k} }}^{z} d_{k}^{z} } \\ \end{array} , $$
  where α_*k*_ and α_*k*_^*z*^ are obtained using a linear search method. The next iteration will be performed until the termination condition is satisfied.

## Wiring design system development

### System design

This system is used to optimize the cable length to guarantee a reasonable bending shape. In a virtual environment with the digital prototype of a product, the cable routing position and fixed position can be determined according to the electrical wiring diagram. The cable length is designed to determine the spatial geometric shape under different complex spatial constraints.

To solve the static model described by Eq. (25), we must know the geometric parameters (length and radius) and material parameters (elastic modulus, shear modulus, and density). Aside from the length, the other five parameters can be easily obtained if the cable type is known. The cable length is uncertain, but the model requires the length as an input condition. To determine this, we proposed a fast method of cable length acquisition based on the spline curve. This method takes the known fixed positions as data points and draws a spline curve based on these. The initial length of the cable can be calculated by integrating the curve along the arc length. Finally, continuous optimization of the length is carried out based on the requirements of the bending radius and spatial layout.

### Fast solution for static model

**Figure 8:**
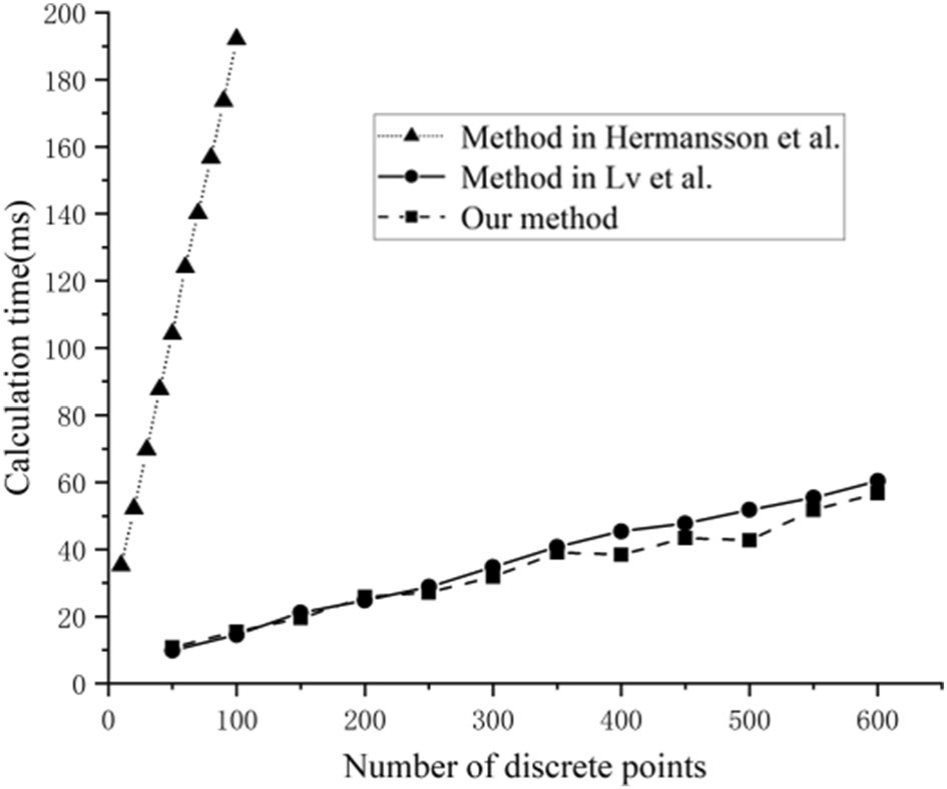
Solution times for numbers of different discrete points.

Figure 8 shows the solution time for different discrete quantities and compares them with those of Ref.^19,20^. The solution time of our method increases approximately linearly with the number of discrete points and is four times more efficient than that of Ref.^20^. Our efficiency is basically the same as that in Ref.^19^, but our model can handle contact constraints and is more complex. Based on the results, the effectiveness of our proposed algorithm and the efficiency meet the requirements of flexible cable digital design.

### Rapid surface modeling

**Figure 9:**
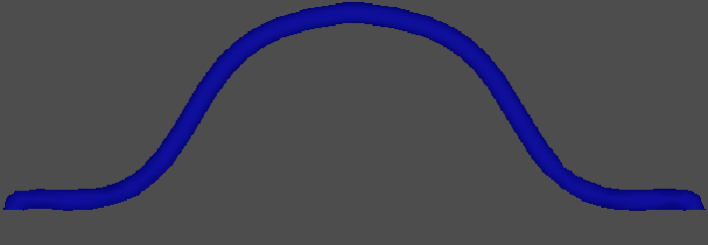
Example of a cable geometric shape.

After obtaining the discrete data points of a cable, the cable surface should be drawn to achieve the geometric shape simulation. The determination of the cable geometric shape simulation usually includes two methods, the curve scanning envelope method and the discrete section connection method^25^. The principle of the former is to first construct a path through the discrete points based on a spline curve and subsequently generate the surface by scanning the cross-section along the path as long as the cross-section orientation is known. The cable surface rendered by this method is fairly smooth, and thus, we selected this method. Figure 9 shows an example of the cable geometric shape.

## Cable routing validation

### Routing module development

**Figure 10:**
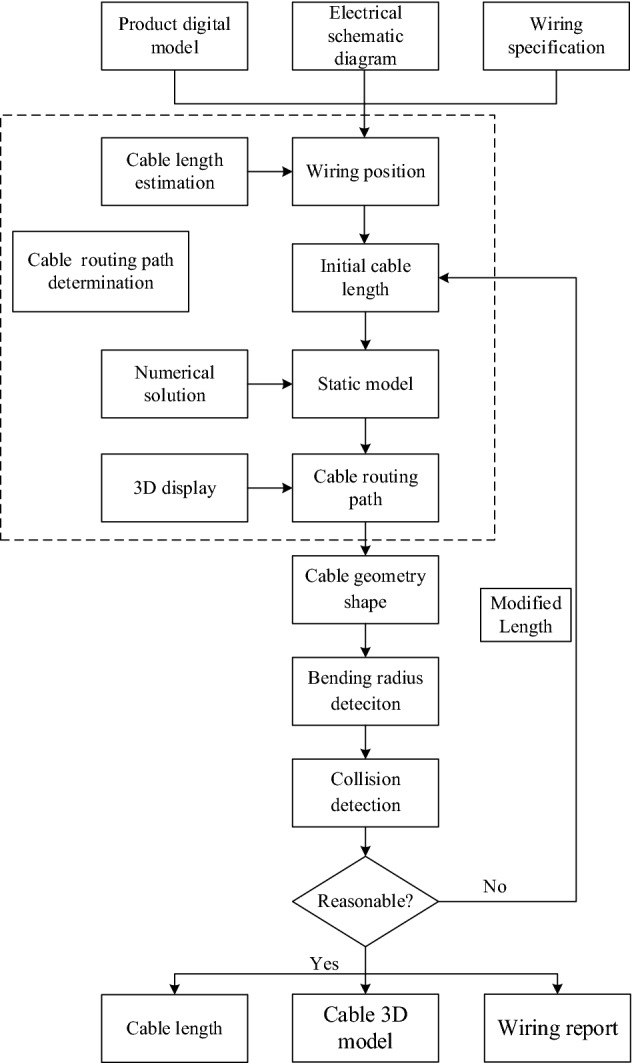
Development process of the cable routing module.

Based on the flexible cable digital design method mentioned in the above section, the cable digital wiring design module is presented in this section. The module development process is shown in Fig. 10.

This module was developed in the Microsoft Visual Studio 2018 (VS2018) environment under the Windows 10 operating system using the C++ language and the existing virtual assembly system^4^. The interface of this module was based on the MFC (Microsoft Foundation Classes) basic class library. The API (Application Programming Interface) provided by AMPL was used to solve the static model. The cable geometric modeling was realized based on an open source geometric kernel system, named Open Cascade (OCC). Data management of the wiring module was implemented using the C++ file operation class fstream.

The cable designer first determines the material use and wiring relationship of the cable according to the digital model of the product and the electrical schematic diagram, after which the key points of the cable are determined based on the wiring specifications. This module automatically provides the initial length of the cable through the position of the key points of the cable and displays the geometry of the cable in three dimensions. The designer can perform interference and bending radius checks. If the results do not meet the requirements, the cable wiring length is amended and rewired until all wiring requirements are met. The module subsequently records the cable wiring path and material usage information. If there is significant interference in the wiring result of a cable within the allowable length range, the cable designer is required to receive feedback from the product design department to optimize the electrical connection position of the product. After completing the wiring design of an aerospace product, the design results are output. The output of the module includes the 3D wiring geometric shape, path information, length, and material usage information.

### Model validity experiment

**Figure 11:**
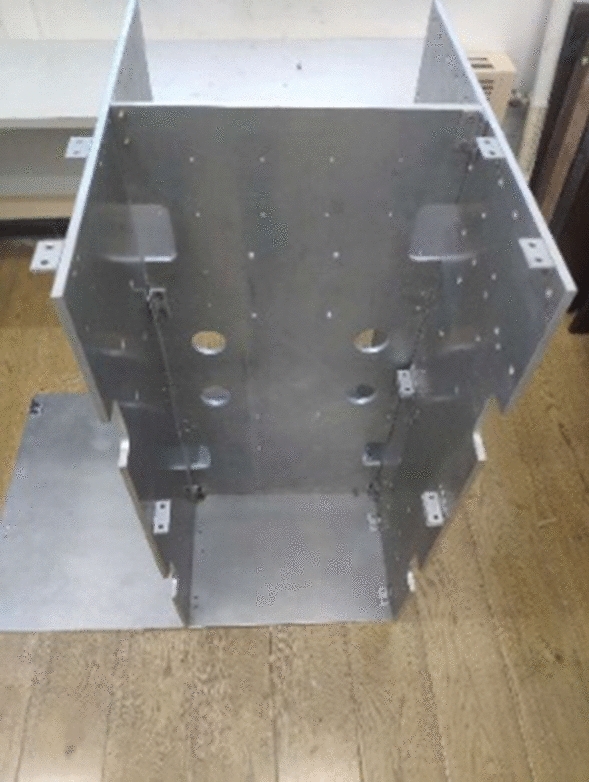
Cable geometric shape test bench.

**Figure 12:**
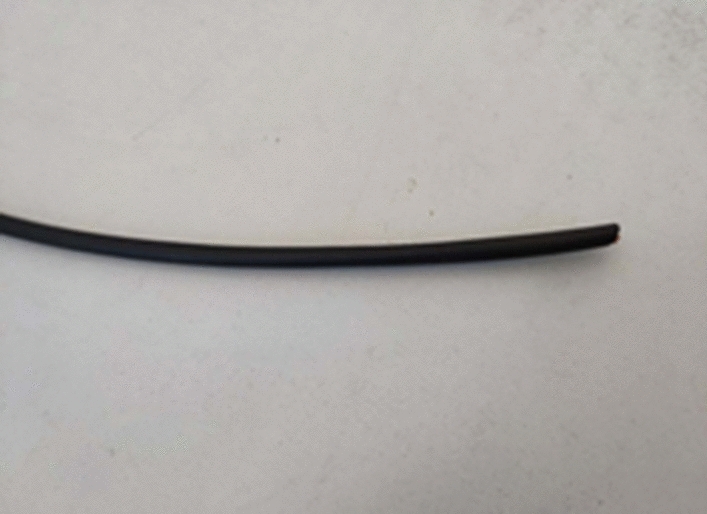
Multi-core copper cable.

To verify the accuracy and validity of our model, a cable geometry test rig was built according to the internal structure model of a satellite, as shown in Fig. 11. There were several holes on the plane of the test bench to simulate the constraints of the passing holes. The two ends of the cable were fixed by the positioning joint. Figure 12 shows a multi-core copper cable. Based on the different lengths of the cables, the cable space shapes were tested under certain boundary conditions, and the results were compared with those obtained by the static model.

The physical parameters and boundary conditions of the multi-core copper cable are given in Table 1.

**Table.1:** Physical parameters and boundary conditions of cable.

Parameters	Value
Radius/m	0.0035
Density/(kg/cm^3^)	1.16
Elastic modulus/MPa	10
Shear modulus/MPa	20
End position/m	(0.16, − 0.235, 0.33)
Left Euler parameters	(0.707,0.707,0,0)
Right Euler parameters	(0.5,0.5., 0.5,0.5)

**Figure 13:**
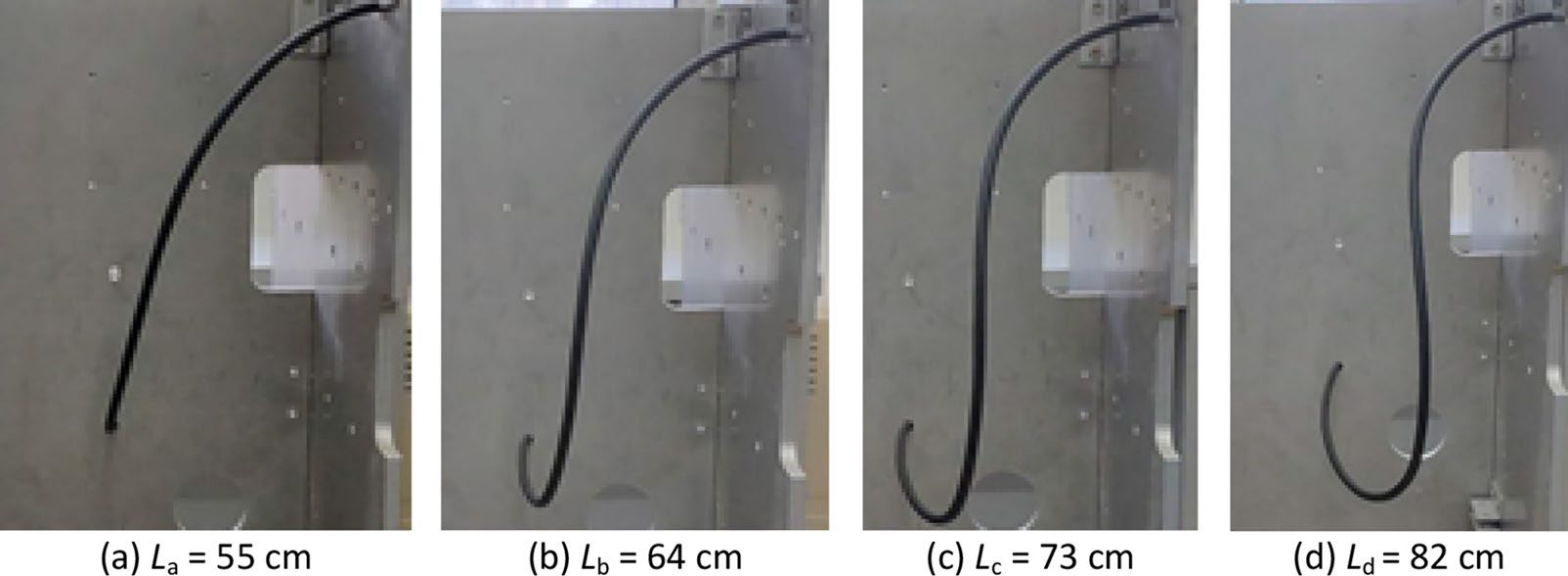
Cable experimental shape change with increasing length.

As shown in Fig. 13a-d, under the same boundary conditions but with different lengths, *L*_a_ = 55 cm, *L*_b_ = 64 cm, *L*_c_ = 73 cm, and *L*_d_ = 82 cm, the bending degree of the cable will gradually increase under the action of self-weight and internal forces.

**Figure 14:**
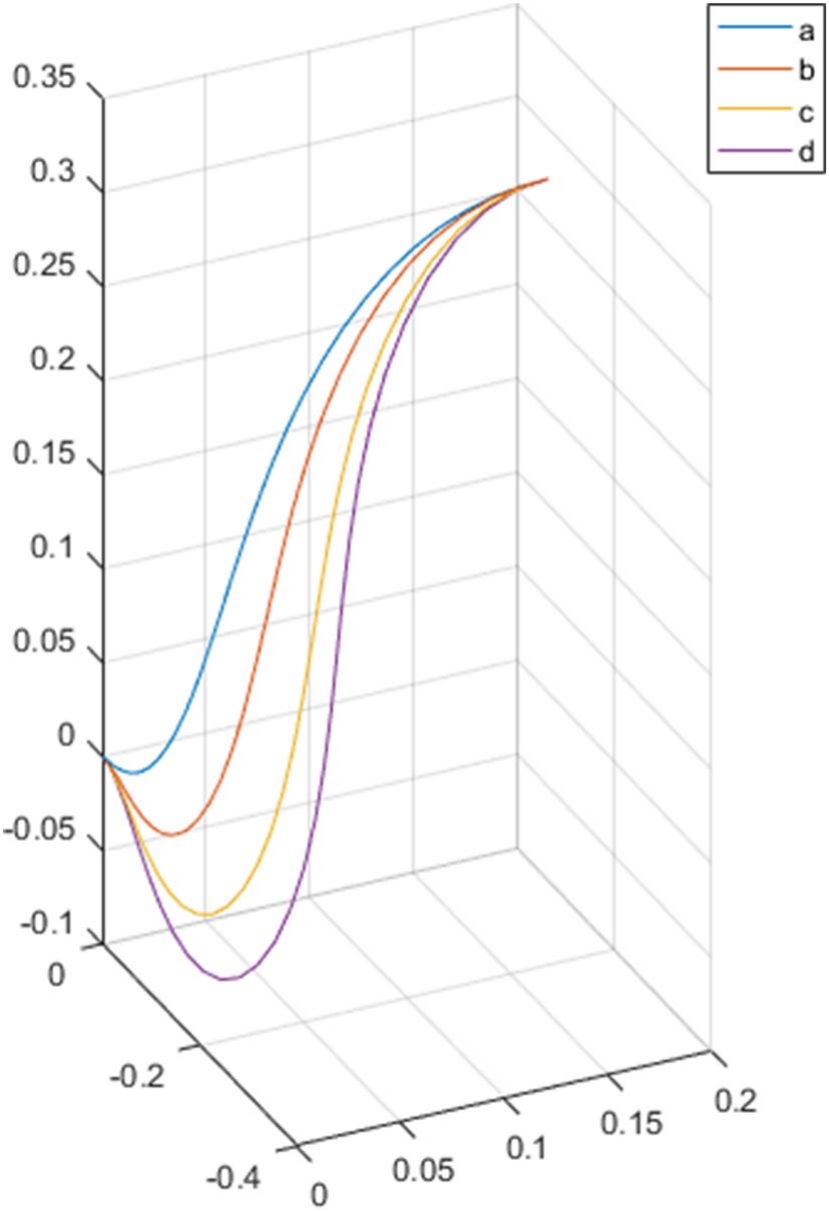
Cable simulated shape change with increasing length.

Using the modeling method proposed in this study, the cable spatial geometric shape was calculated. As shown in Fig. 14, the shape of the cable centerline was drawn in Matlab. The simulated cable curve was consistent with the experimental results, which proved the validity of the proposed model and algorithm.

### Application cases

**Figure 15:**
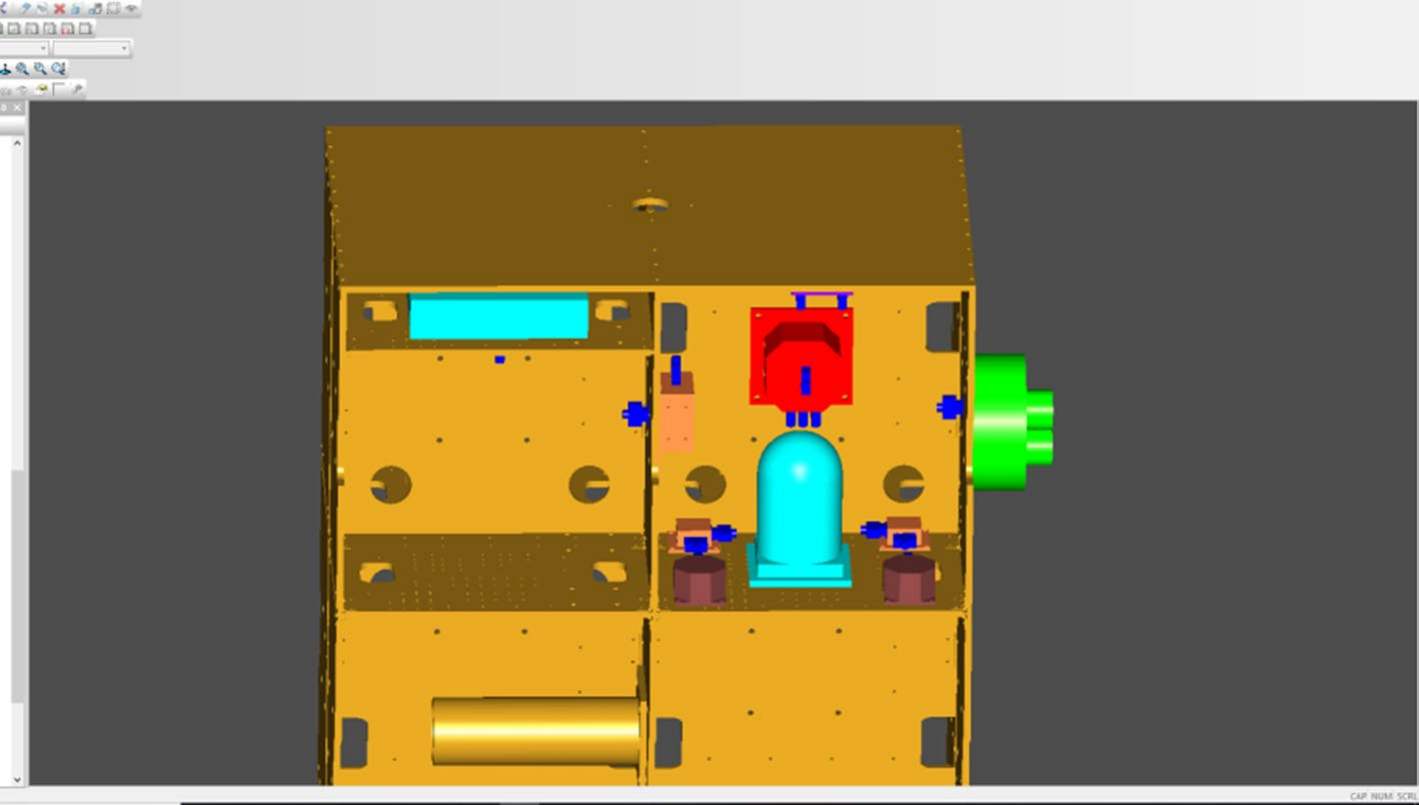
Digital prototype of the satellite simulator in our wiring system.

**Figure 16:**
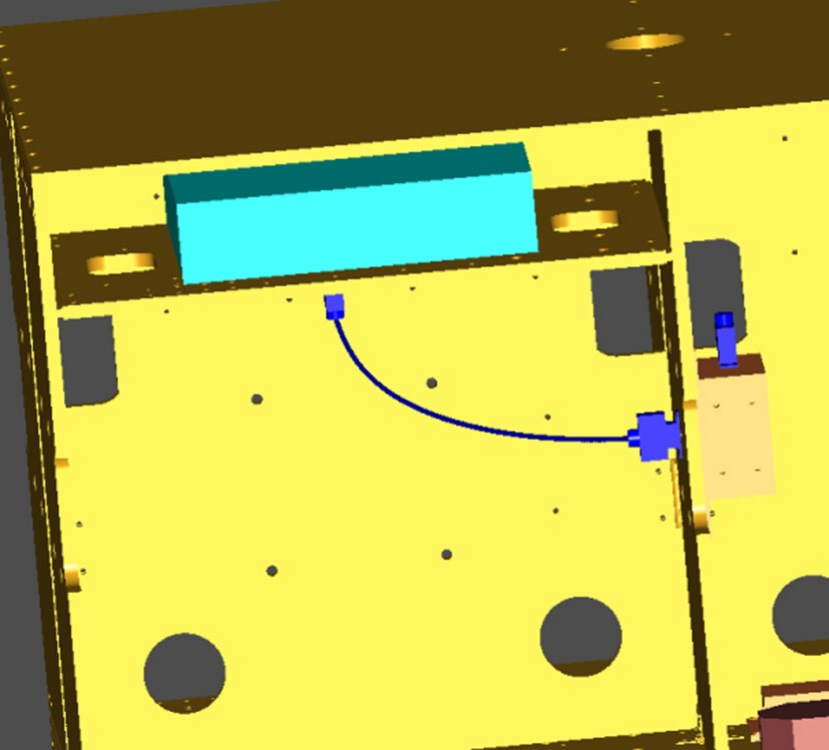
Single cable wiring.

**Figure 17:**
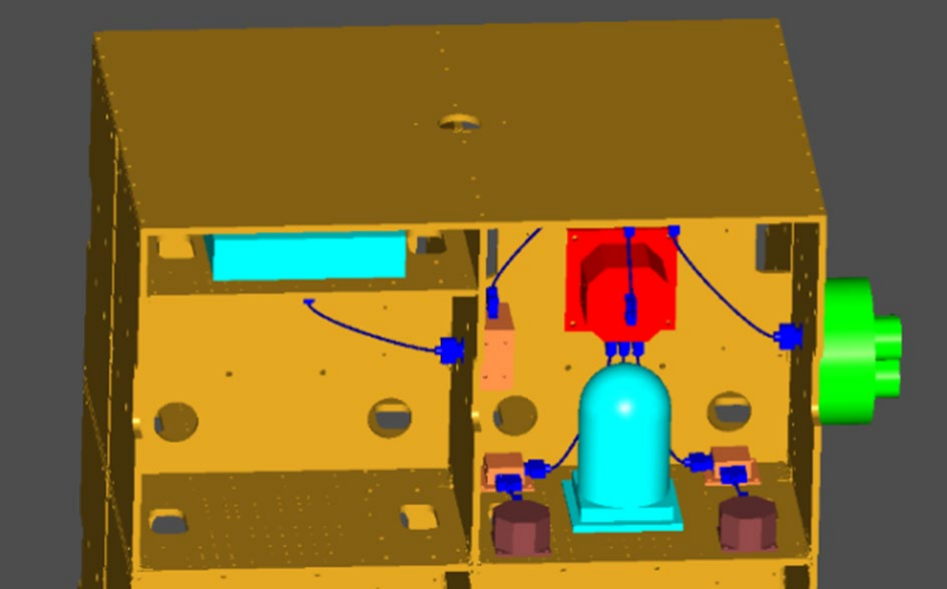
All routing cables in the wiring module.

To further illustrate the validity of the flexible cable digital design method proposed in this paper, the functions of the developed cable digital wiring design module were introduced with a wiring process of a satellite simulator, and the relevant results were verified. The steps were as follows:

1. In a 3D CAD environment, based on the requirements of the electrical connection and wiring experience, the cable connector model was determined, and the products and cable connectors were assembled. The assembly model of the satellite simulator was transformed into the model data format (.step) required by our wiring system. Figure 15 shows the 3D model of the satellite simulator.
2. According to the requirement of the electrical schematic and wiring diagrams, the cable materials were determined, and the material library was established.
3. First, a cable was wired. The cable name was established through the new cable interface, and the material name was selected to establish the cable. In the constraint management interface of the system, the circular section of the cable connector was selected as the constraint surface, and the direction constraint of the cable connection was defined.
4. By choosing the appropriate cable connector using a mouse to build the cable, the program completed the calculation of the cable static model and generated the cable surface in real time. Figure 16 shows the result of the single cable wiring.
5. The bending radius of the generated cable was tested. When the bending radius exceeded the user's set value, the length of the current cable was acquired and modified to facilitate re-wiring.
6. Steps 1–5 were repeated until all cables were wired. During the wiring, the interference was monitored at all times. When the interference occurred, the cable length was changed and rearranged. Figure 17 shows the results of the 3D wiring after all cable designs were completed.

### Digital wiring verification

**Figure 18:**
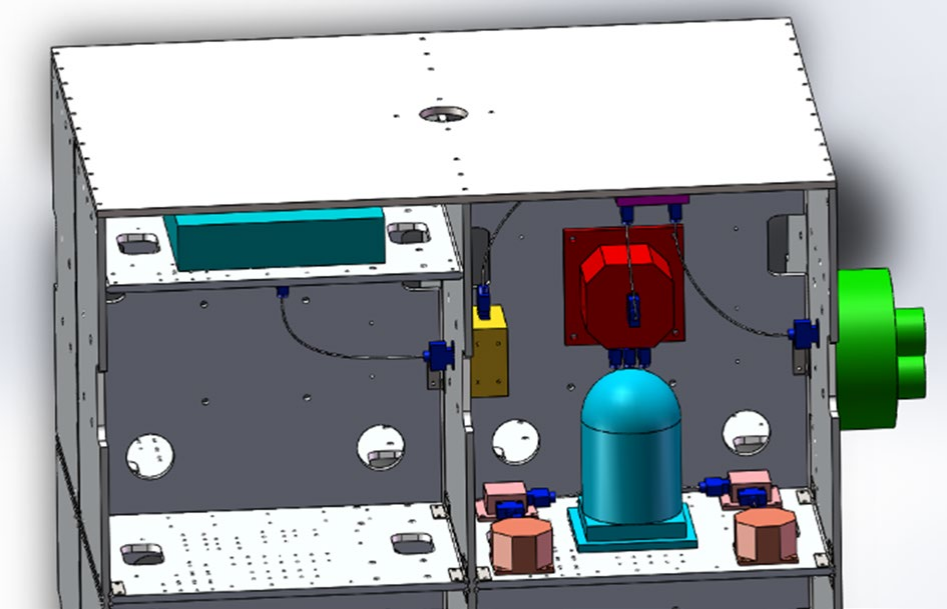
Routing cables in SolidWorks.

To illustrate the correctness of the above wiring case, further comparative verification was carried out. First, combined with the digital prototype of the satellite simulator, wiring was carried out in the wiring design plugin provided by the SolidWorks software. By selecting the end circle section center of the cable connector as the connection point of the cable, wiring was carried out using the automatic wiring function. The wiring result in SolidWorks (version: 2019 SP5.1; URL: https://www.solidworks.com) is shown in Fig. 18.

**Figure 19:**
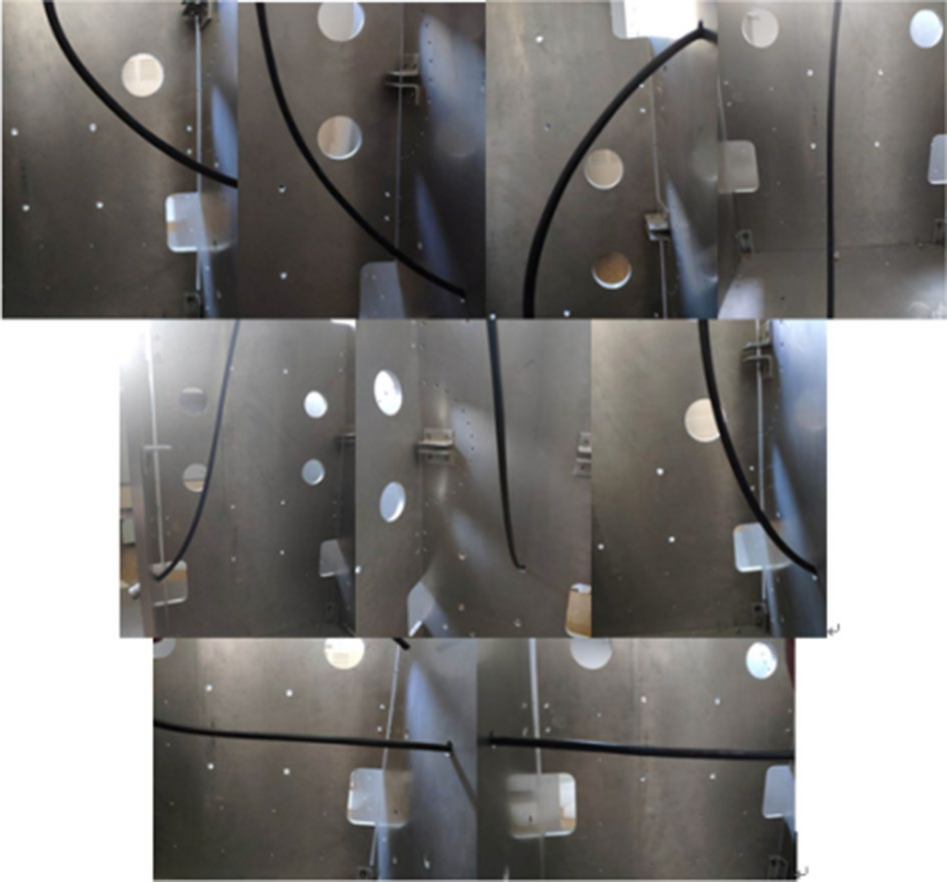
Routing cables in the test bench.

The length of each cable was determined by the SolidWorks measurement function. Meanwhile, wiring experiments were carried out on the test bench. The wiring results were recorded using a camera, and the results are shown in Fig. 19.

The wiring results from SolidWorks and the wiring shape test bench were compared in the flexible cable digital wiring design module. Comparing the wiring results in the three environments, as shown in Table 2, the average wiring length of the proposed method was 12.2 mm longer than that of the wiring test bench, corresponding to a difference of 7.25%. The wiring length in SolidWorks was 24.98 mm longer than the actual result, and the difference was 13.55%. The results showed that the design length of the cable decreased by 6.3%. The digital design method of the flexible cables proposed in this paper could effectively reduce the cable design length and realize the lightweight design of products.

**Table 2:** Length comparison of wiring results.

Cable	Wiring module/mm	SolidWorks/mm	Test bench/mm
1	238.79	266.24	221
2	196.51	212.80	173
3	145.26	153.60	139
4	218.39	233.55	201
5	203.26	221.38	196
6	278.90	296.01	271
7	189.41	204.16	168
8	77.92	76.73	73
9	83.46	82.39	80

## Conclusion

In this study, a computer-aided optimal design method for flexible cables was proposed based on a variational principle. Optimization of the geometric parameters, such as the length and bending radius, of flexible cables in aerospace products was achieved. The problems caused by the wiring design of existing CAD software, such as the large product quality, uncertain centroid, and poor reliability, were resolved. The main conclusions were as follows:

1. The rapid solution of the cable geometry was achieved. The algorithm was developed by AMPL and Visual Studio, which guaranteed its efficiency. Our solution speed was four times greater than that of Ref.^20^. Our efficiency was similar to that of Ref.^19^, but our model could handle contact constraints and more complex shapes. From the results, the effectiveness and efficiency of our proposed algorithm met the requirements of flexible cable digital design.
2. The function of the digital wiring module was verified by an example, and the wiring and experimental results were compared and analyzed. The results showed that the design length of the cable decreased by 6.3% to achieve the minimum bending radius.
3. From the above analysis, the model and algorithm proposed in this paper could effectively solve the problem of inaccurate design parameters of the cable (large length and bending radius), and it could handle a variety of complex constraints. The algorithm presented by Ref.^20^ could only simulate the cable shape based on the boundary conditions. While the algorithm presented in Ref.^20^ could find a reasonable route, it could not optimize parameters such as the length and bending radius. Ref.^19^ solved the problem of branch wiring from an optimization perspective. However, it could not account for contact constraints and optimize the length and bending radius.

In summary, compared with traditional commercial design software, the flexible cable digital design method proposed in this study can effectively reduce the length of cable design, increase product quality, and improve the reliability of aerospace product design. It lays a theoretical and technical foundation for effectively solving the problem of a large error in the cable manufacturing and achieving lightweight designs of products. However, the algorithm should be further expanded and studied as follows:

1. Efficient and robust cable dynamics modeling and a numerical calculation method should be studied.
2. Computer-aided design and assembly of flexible cable bundles should be developed.

## Data Availability

All data generated or analyzed during this study are included in this published article.
